# CORRELATES OF CANNABIS USE AMONG MIDDLE-AGED AND OLDER ADULTS: FINDINGS FROM NESARC-III

**DOI:** 10.1093/geroni/igac059.2839

**Published:** 2022-12-20

**Authors:** Kevin Yang, Jaclyn Bergstrom, Alison Moore

**Affiliations:** UC San Diego School of Medicine, San Diego, California, United States; UC San Diego School of Medicine, San Diego, California, United States; University of California, San Diego, La Jolla, California, United States

## Abstract

Cannabis use is increasing faster among both middle-aged and older adults compared to younger adults, but its demographic and physical health correlates comparing middle-aged and older adults need further exploration. We examined data from a US representative sample of middle-aged (50–64 years, Nf8,932) and older (65+ years, Nf5,806) adults from the 2012–2013 National Epidemiologic Survey on Alcohol and Related Conditions (NESARC-IIII). We conducted logistic regression analyses to test associations of cannabis use with demographic and past-year physical health correlates and examined differences between the two age groups. An estimated 5.6% of middle-aged and 1.3% of older adults used cannabis in the past year. Compared to middle-aged adults, older adults had higher rates of cannabis use for medical purposes (15.8% vs. 12.3%, p=0.033). Both age groups had increased odds for being male (Odds Ratio (OR)=2.32 and 2.63, for middle-aged and older adults, respectively) and residing in the West (OR=1.82 and 3.04, respectively). Middle-aged cannabis users were at decreased odds for having at least some college education (OR=0.63), income >$70,000 (OR=0.37), being married (OR=0.32), and reporting excellent/very good general health (OR=0.65). Middle-aged cannabis users were at increased odds for reporting digestive disease (OR=1.96), musculoskeletal pain (OR=1.34), and nerve pain (OR=1.77), while older cannabis users were only at increased odds for reporting digestive disease (OR=2.91). Findings indicate differences in cannabis use correlates between middle-aged and older adults. With increasing legalization of cannabis nationally, improved understanding of these correlates among more recent cohorts will assist in monitoring cannabis use among the older adult population.

